# Hospital Reports

**Published:** 1837

**Authors:** 


### WESTERN JOURNAL

OF THE

### MEDICAL AND PHYSICAL SCIENCES:

**For April, May, and June, 1837.**

### ESSAYS AND CASES.

### Art. I.—Hospital Reports.

*Cases Treated in the* Cincinnati Hospital, *with Clinical Observations.* Recorded by William J. Barbee, M. D., and Edward Kimball, Student of Medicine, House Surgeons. Arranged by the Editorial Committee.

The Cincinnati Hospital was established, in the Autumn of 1836, by the Professors in the Medical Department of the Cincinnati College, for the instruction of their pupils in clinical medicine and surgery. The building selected for the purpose, stands directly opposite the college edifice, and will accommodate about 30 patients. By a contract with the General Government, it is a Marine Hospital, so far as there are sick boatmen at this place; and by an arrangement with the Surgeon of the *Cincinnati Eye Infirmary*, an incorporated charity, the patients of that establishment are received and treated in the Hospital. Other patients, moreover, are admitted, both as beneficiaries and by paying for their accommodations. Since the house was opened, on the first of October last, it has never been without patients, and at times the number has been such, as to afford many decided practical advantages to the pupils of the College. All who intend to graduate, are required to take the hospital ticket, and attend the practice throughout their second course of lectures. During the session, the professors of surgery and of the theory and practice, attend to the sick, daily, from two to three o'clock in the afternoon; thus affording to the medical students an opportunity of witnessing nearly every prescription made, without losing any of the lectures in the College

In the infancy of such an establishment, its reports cannot conveniently display that fulness and circumstantiality, which are the fruits of an older organization; but, in the subsequent numbers of our Journal, we hope to present such as will, in their details, be found of deeper interest.

### SURGICAL DEPARTMENT.

### CASES OF SCALDING BY STEAM.

Before day, on the morning of the 17th of November, 1836, the steamboat Flora, on her upward voyage, about forty miles below Cincinnati, suffered a fracture of her equilibrium pipe. The steam escaped in large quantities, and entered the door of the gentlemen's cabin, which was standing open. The passengers were aroused from their sleep, and ten or twelve of them were more or less extensively scalded. One died before reaching our quay, and another was *in articulo mortis* when the boat arrived. Most of the others became patients—internal or external—of the *Cincinnati Hospital.*

On examining them, it was found that no part of their bodies that were covered by clothing, however thin, were scalded in the slightest degree. Thus it appears, that steam does not act injuriously through our clothing, and that all who travel in steamboats should be careful, at night, *to lie down clothed.* In further testimony of the same fact, it may be mentioned that such of the passengers as remained in their berths, and covered up their heads, escaped all injury.

When the sufferers reached Cincinnati, reaction had taken place in most of them, as they had been liberally supplied with brandy and laudanum; but the pain and smarting which they still experienced, were very great.

Case.—Col. K., a member of Congress from Indiana, was among the number of those who were scalded most severely. Nearly the whole of both hands and both feet, with his wrists and ankles, and his face and neck, were blistered; the other parts of his body were covered. It did not appear, that the mucous membrane of his mouth, throat, lungs, nostrils or eyes, was in the slightest degree affected, although the cuticle was raised to the very margins of the different apertures. Hence, it seems that steam does not act on the mucous membrane as on the skin.

The dressings selected for this patient, by Professor Drake, was the common liniment of flaxseed oil and lime water. When he arrived, his pulse appeared to be well sustained, but it was soon found to be variable and occasionally to sink—rendering a repetition of the laudanum necessary, not only to relieve his sufferings, but to support his circulation. His mind was agitated and alarmed; and in the course of the first night, he showed signs of delirium. On the succeeding day, the laudanum was replaced by a solution of the acetate of ammonia with acetate of morphia, but no particular advantage followed the change. On the third day, the mental excitement was great and disordered. At times he would breathe, in imitation of the puffings of a steamboat; and his exclamations indicated that his mind was running on the scenes of disaster and distress, connected with the explosion by which he had suffered. His pulse was frequent and sometimes tense. The scalded surfaces, red and tumid. In this condition, to relieve his brain, he was bled to 12 or 14 ounces. His pulse sank, and the denuded surfaces assumed a darker hue, but no abatement of the encephalic disorder followed. Copious purging was effected, but without any decided advantage. The dressings were now changed for those of a more stimulating character; consisting of the Kentish or terebinthinate liniment. A copious secretion of laudable pus followed this application, with an abatement of the cuticular tumefaction, and an appearance of healthy granulation; but his constitutional symptoms continued bad. His pulse became more frequent and variable; his *conjunctivæ* reddened; a considerable degree of convulsive action of his muscular system, with great restlessness supervened; and his aberation of mind remained unmitigated. As the vital powers were sinking, yeast poultices with cinchona bark, were applied to his extremities over the liniment, and continued up to his death, which took place about six days after the accident—his delirium remaining to the close of life. Dying at the house of a friend, a *post mortem* examination was not made.

Case.—S. D., of Washington, Pa., aged 27, was admitted into the Hospital at 4 o'clock, on the 17th of November. twelve hours after the accident. Scalded over his entire face, forearms and hands, abdomen and feet and legs—having gone into his birth with no other clothing than a shirt. Some reaction in his general system had taken place. On board the boat, his lower extremities had been covered with rags dipped in a liniment of white lead ground in oil. To the other parts, Professor Parker applied the liniment of flaxseed oil and lime water, and ordered sixty drops of laudanum. At 9 o'clock, P. M., his pulse was seventy in a minute and rather weak. His tongue furred.

18th. The same applications were continued, and the saturnine liniment left on his legs. Pulse 110; tongue covered with a brown coat; bowels costive. An enema was ordered, which produced two or three evacuations of a healthy character. At midnight his pulse was 112, and he was extremely irritable: ordered sixty drops of laudanum.

19th. Rested well the latter part of the night. In the morning, pulse 108, with strong signs of constitutional irritation. Took ℥j of ol. Ricini, and had several operations through the day of a bilious color. The same applications were continued, and forty drops of laudanum administered at night.

20th. Rested well last night—pulse 98. Applications the same. Laudanum at night.

21st. Passed a tolerable night—pulse 112—costive. Ordered sulphate of magnesia and soda water. Had a copious operation at 8, P. M. After which he took a portion of Dover's powder and calomel; dressed with the Kentish liniment, covered with varnished silk.

22d. Pulse 116. Lies in a half comatose state. Ordered a pill of opium and tartar emetic—each, one grain. In the afternoon, pulse feeble; directed a grain of sulphate of quinine and half a grain of opium every four hours.

23d. Considerable delirium last night, his circulation being extremely feeble. Died at 5 o'clock, this morning.

Post mortem examination, *five hours afterwards, by Professor Gross, in presence of the Faculty and Students.*

Skin. No gangrene had taken place, but there was pus on the scalded surfaces. Underneath there was considerable cellular infiltration.

Brain. Consistency natural. Great venous congestion. Bubbles of air in some of the superficial veins. Some adhesions between the hemispheres. A sero-purulent effusion between the dura mater and arachnoid.

Lungs. Engorged with blood. Mucous surface up to the glottis, in a state of hyperæmia, with appearance of purulent secretion.

Heart. Natural, but a considerable degree of hydro-pericardium.

Organs of the Abdomen. Stomach healthy, except two spots of sanguinous extravasation underneath the mucous coat. Intestines distended with gas, otherwise natural. Liver congested. Spleen adherent to the diaphragm, and much engorged with blood. Bladder, with a quart of urine. It was supposed that the patient had discharged his urine during the operation of his cathartics and injections, as he made no complaint of its retention; and being scalded in the hypogastric region, the state of his bladder was not ascertained by an examination.

Case.—G. F., aged 39, a robust German, scalded on the face, breast, shoulders, arms and hands, was admitted at the the same time. Pulse 60. Constitutional irritation and prostration not so great as in the other cases. Placed in the immediate charge of Mr. Trask, one of the clinical pupils. Professor Parker ordered the same dressings as in the case of S. D. just narrated.

18th. Rested badly during the night. Left hand livid, and the circulation in the whole limb very inactive. Applied the Kentish liniment. Pulse 80. Tongue with a brown coat.

19th. Slept better. Purulent secretion general, except on the left hand, the back of which showed strong signs of gangrene. Continued the liniments, applying to the left hand a fermenting poultice. Opium and tartar emetic mixed in grain doses, each.

20th. Pulse 80. Rested well last night. Same dressings continued. Cream of tartar administered as a laxative.

21st. Pulse 80. Slept well last night. Suppuration on the left hand begun. Poultice continued. Circulation well sustained. Professor Parker now directed, to the scalded surfaces generally, charpie dipped in cold water, to be covered with medicated silk.

22d. Pulse 80. Doing well. Gangrenous skin and cellular tissue of the left hand sloughing off. Treatment the same.

23d. Not so well. Rested badly last night. Slight delirium. Opium and tartar emetic, each in grain doses mixed, through the day. At night, being costive, 10 grains of calomel, and 10 of rhubarb.

24th. Decidedly better. Recovery from this time regular and rapid.

Case.—T. J., of Steubenville, aged 18 years, admitted at the same time, with the other patients. Scalded on the face, arms and hands. Pulse 58. Has an enlarged spleen, from intermittent fever. The calcarious liniment applied as in the other cases ; and 50 drops of laudanum administered.

18th. Rested very indifferently last night. Great constitutional irritation. Pulse 112. Dressings the same. Calomel, 4 grs. Ipecac. 1 gr. mixed.

19th. Slept a while last night. Still very irritable. Two or three alvine discharges of a healthy character. Pulse 116. In the evening, pulse 104, restless. Ordered opium and tartar emetic. Emollient poultices to his hands.

20th. Rested well last night. Took calomel and rhubarb. Same dressings continued. In the afternoon took Ol. Ricini.

In the evening had two operations. Directed opium and tartar emetic at bed time.

21st. Pulse 92. Rested better last night. Same dressings continued. Purulent secretion established. Dressings—wet lint and medicated silk. Laudanum at bed time.

22d. Slept well last night. Pulse 76. Same dressings, except to his hands, which, being in pain, were covered with emollient poultices. Ol. Ricini in the day, and opium and tartar emetic at bed time.

23d. Symptoms favorable. Pulse 75. Applications the same. At night, opium, tartar emetic, and calomel.

24th. Symptoms less favorable. Pulse 85. Slight delirium. Cathartic of Ol. Ricini. Evening—the medicine has operated well. Better.

25th. Still better, but irritable in the evening, with his pulse at 82. Twenty drops of laudanum.

From this time, he continued to recover regularly, and was discharged on the 4th of December.

The treatment of the other patients was essentially the same as that which has been detailed, and they recovered.

### Clinical Remarks by Professor Parker.

In his practical remarks on the applications appropriate to injuries from caloric, the professor drew a distinction between burns and scalds. A very *limited* scald and a *superficial* burn, may be treated in the same manner. A simple emollient or a refrigerant,—a bread and water, or an elm bark, or a carrot poultice,—or a compress dipped in lead water,—will be sufficient, and equally adapted to both cases. It is when the injury is severe, that a necessity for some variety in the topical applications arises. The reduction of the vital powers of the part, in deep burns, is very great, and the dressings from the beginning should be stimulating. It was in cases of this kind occurring in the mining districts of England, that Mr. Kentish first used his turpentine liniment. Tar is an excellent application to such burns. An emollient poultice should be applied over these stimulant dressings. Early suppuration is the desideratum. If the state of excitement which attends purulent secretion, should not arise in time, the part will lose its vitality. Scalds are dangerous in proportion to their extent. The disease is confined to the skin, which is exquisitely sensible ; and brings into sympathy a great number of the vital organs. Bad scalds, therefore, do not require applications so stimulating as those adapted to bad burns. Of every kind of dressings, Professor Parker prefers the liniment of flax seed oil and lime water. It is slightly stimulating, emollient, and calculated to exclude the atmosphere, the action of which on the skin denuded of its cuticle, is always injurious. It is only when the surface changes from a bright to a dark red, indicating passive congestion, with but little tendency to puriform secretion, that the Kentish liniment, and other exciting applications, are required, or even proper. To this remark, however, exceedingly superficial scalds, not amounting to vesication, are an exception ; as such are often successfully treated with whiskey and other alcoholic lotions, which seem to introduce a new action into the part. The Professor doubts the propriety of applying liniments of white lead extensively, to parts denuded of their cuticle. They may act on the nervous system as poisons. In the granulating stages of scalds, he has a very favorable opinion of lint dipped in cold water ; provided it is covered with oiled or varnished silk, to prevent evaporation and exclude the air. It is acceptable to the feelings of the patient, and favorable to early and healthful cicatrization.

### Further Remarks by Professor Drake.

At the close of the treatment of these cases, the Professor took occasion to point out to the pupils some of the difficulties attendant on the constitutional treatment of extensive scalds. The nervous function is prostrated ; not, however, by a narcotic, but an agent whose ultimate or secondary action is, to produce inflammation and a phlogistic diathesis. Therefore, while the vital powers are greatly reduced, there is a tendency in the central organs to inflammation. This tendency is greatest in the brain. Hence the symptoms of phrenitis, in the patients whose cases have been narrated ; and the actual existence, on dissection, of the products of inflammation in the case of S. D. The professor observed, that from the very nature of the injury, such cases would always be liable to a sinister termination. If patients were over stimulated, they would certainly die of acute inflammation—if depleted, they would sink. Depletion could not reach the inflammation, while the prostration of the nervous power continued; and blood-letting was apt to increase and prolong the constitutional irritation, even when it did not occasion death. In this combination of irritation and inflammation, he supposed the best remedy is the compound of opium and tartarized antimony, in a solid form—the latter being used in large doses, after the manner of the Italian physicians. Sometimes full vomiting, followed by an anodyne is beneficial.

### Caries of the Bones of the Foot. Amputation.

Case.—Jolly, aged 30 years, was admitted into the hospital on the 14th day of December, 1836, for disease of his left foot. His constitution was good, and his habits temperate; but he was considerably emaciated, without, however, being decidedly hectical. About fourteen years before, his heel was frost-bitten, and there followed some internal inflammation, with the discharge of some spiculæ of bone. This disease seemed, however, to get well; and he had the perfect use of his foot and limb till about two years before the time of his application at the hospital; when, without any obvious cause, the parts under the ankle joint, became affected with pain and swelling, followed by several fistulous openings and an ichorous discharge. Having tried various plans of treatment, he was received into the hospital.

Professor Parker, to whose care the patient was confided, concluded that the only chance of relief was to remove the limb, and he accordingly, on the same day, performed the operation in the anatomical amphitheatre, in the presence of the medical class, cutting off the leg about four inches below the knee. In the course of four weeks the stump was so far healed, that Jolly was able to leave the hospital, and with the aid of a cork leg, made by some artizan of this city, and he has no difficulty in attending to his ordinary pursuits.

On inspecting the amputated limb, Professor Gross found the astragulus and os calcis quite carious, with a distinct abscess in the centre of the former, filled with greenish looking and highly offensive pus; of which a considerable quantity had been latterly discharged through the fistulous tracks before alluded to. The subcutaneous cellular tissue and skin of the instep and sides of the foot were of a dense gristly hardness in different parts, and the fistulous passages lined by a thick layer of lymph. The other tarsal bones were perfectly sound.

The posterior tibial nerve, in this case, was very much enlarged, being at least three times the natural size, and its sheath was covered for a considerable distance with hardened lymph. The corresponding artery was ossified and very brittle.

Five weeks after the removal of the limb, Jolly still complained of severe pain in the sole of the foot and along the metatarsal bone of the little toe; parts in which he suffered much distress previously to the operation. This symptom is by no means unusual. Count De Sigur states that it was very common in Buonaparte's soldiers, who were obliged to submit to amputation during his disastrous campaign in Russia.

### Fatal Psoas Abscess. Dissection.

Case.—On the 19th of October, S. M. B., aged 27 years, was brought to the Hospital from Fairfield county, Ohio, with disease of the spine. When a child, as he stated, he had a slight curvature of the lower part of the dorsal spine. For the last ten years, his health had been bad, and about five years before the time of his admission, the distortion of his spine suffered a considerable increase; a year or two after which, collections of matter began to point, and were punctured by his physician. On being examined, five fistulous openings were found. One directly under the ilio-pubic ligament; one on the under and upper part of the left thigh; one over the gluteus maximus, and two others on the trunk of his body. The discharge from them was a tolerable pus. He had hectical symptoms and was much emaciated. On the 22d, Professor McDowell introduced two setons, over the protuberant vertebræ, and he was subjected to therapeutic treatment. On the 18th of May, 1837, he died, having been subjected, by Professor Parker, to long continued but mild courses of mercury, iodine, cicuta, sarsaparilla, and bitters; with injections of creosote water, and a solution of nitrate of silver. At times, during his protracted sojourn in the hospital, his general health improved, and he regained some of his lost flesh; but none of the fistulæ closed up, and towards the last, the discharge from them became extremely offensive. It is not necessary to give the details of the treatment, as the case is interesting chiefly for its pathological anatomy. Twelve hours after death, he was examined by Professor Gross, who reported the following morbid appearances:

External Aspect.—Body much emaciated; knees rigidly flexed; legs and feet œdematous, the right more than the left.

Brain.—Membranes sound, and also the cerebral substance, excepting the septum lucidum and fornix, which were much softened, the former almost indeed destroyed; spinal cord natural.

Thorax.—Both lungs every where adherent to the walls of the chest, by ancient cellular tissue, the attachment being strongest along the sides of the spinal column, where it was effected by a greyish adventitious membrane, of a dense fibrous structure, about the twelfth of an inch in thickness, and perfectly organized. Substance of the lungs sound, and without a single tubercle. Pleuretic sac projected on each side fully two inches above the clavicle. Air tubes natural. Some of the bronchial glands calcarious.

Heart.—Somewhat flabby, but otherwise natural. Aorta and great veins sound.

Abdomen.—Omentum considerably wasted; rest of the peritonæum sound, and its sac containing about six ounces of thin straw-colored fluid.

Œsophagus, stomach and intestines perfectly sound throughout.

Liver of the natural color, size and consistence; but here and there was found a small osseous concretion, of a yellowish hue, varying in volume from a pin head to a currant, altogether about fifteen in number. Most of them were distinctly encysted, the capsule, which was of a greyish color and fibro-cartilaginous firmness, being about the fourth of a line thick, and closely adherent to the hepatic tissue, which itself was quite sound. Gall-bladder healthy, and filled with black, thick, ropy bile, like treacle.

Spleen nearly twice the usual bulk, and somewhat indurated, with several small patches of ancient lymph on its exterior. Near its centre, internally, was found an osseous concretion, similar in appearance and volume to those of the liver.

Pancreas sound.

Lumbar and sacral lymphatic glands much enlarged, extensively tuberculated, infiltrated with serosity, and many of them of a light slate color. A considerable number of them contained osseous concretions, mostly of a spherical shape, about the size of a mustard seed, isolated, hard, and of a yellowish color. In one of these glands there were nearly as many as twenty of these little bodies, of an irregular shape and closely agglomerated.

Kidneys.—The right, of the ordinary size, adhered firmly to the surrounding parts by indurated cellular tissue. The capsule was opake, and slightly thickened, especially in the lower half of its extent; and the outer surface of the organ, of a red mottled aspect, was studded with upwards of five hundred tubercles, some as large as a cherry-stone, others about the size of a mustard seed. In some parts they were agglomerated, in others isolated. In the presence of my colleague, Professor Harrison, and several office-students, I cut some of these tubercles through at the centre, and satisfactorily demonstrated the existence of vessels, the blood standing upon the incised surface in very minute dots.

In the interior of the kidney were two tubercular excavations; one, situated in the superior extremity of the gland, was not larger than a hazel-nut; but the other, which occupied the lower half of the organ, was about the size of a turkey's egg, and filled with thin, ropy, whitish pus, destitute of smell. The abscess was lined by a thick layer of lymph, of a greyish color and firm consistence; and intersecting it in different directions, were three or four rounded cords, the remains, probably, of the tubular tissue, which resembled, a good deal, the fleshy columns of the heart, or the bands which we so often see in tubercular excavations of the lungs. The interior of the upper half of the glands was of a pale rose color, and very dense in its texture, with here and there a small tubercle.

The right *ureter*, which adhered firmly to the surrounding parts, by dense cellular substance, was of a hard gristly consistence, upwards of half an inch in diameter, and almost closed from one extremity to the other, by a thick lining of lymph.

The right *supra-renal capsule* was remarkably hard, of a greyish mottled aspect, and filled with concrete tubercles, many of them quite large. The left kidney and ureter were natural; but the supra-renal capsule was of a light slate color, and contained several large tubercles.

The *urinary bladder*, scarcely larger than that of an infant, contained about two ounces of thick purulent fluid, resembling that which was found in the right kidney. The mucous coat round the mouth of the urethra, over an extent of about an inch and a half in diameter, was ulcerated. Most of the ulcers were of a circular shape, with reddened, elevated, and slightly undermined edges, and did not reach further than the submucous cellular tissue. The largest was an inch in length, by half an inch in width; the smallest about the size of a split pea. In several of these erosions there was an evident effort at reparation, as was indicated by the strongly adherent lymph which was stretched across their face, and which gave their surface a rough, irregular appearance. Every where else the mucous tunic was perfectly sound. Could these ulcers have been occasioned by the irritating nature of the pus which must have been lodged, from time to time, in this organ?

The Seminal Vesicles were less than one-third the natural size, with very thin, flesh-colored parieties, and filled with curdy, scrofulous matter, of a light greyish tint. The ejaculatory ducts were closed with a similar substance. The left deferential tube was impervious and very gristly; the right was filled with tubercular matter. Some of the follicles of the prostate contained the same kind of substance; but in other respects, the gland was natural.

The right External Iliac Vein, as far as its union with the ascending cava, the internal iliac, right spermatic, vesical, and femoral veins were thickened, some of them very much, and closed with black coagulated blood, which adhered firmly to their lining membrane by processes of lymph. On wiping away this matter in different places, the internal tunic was found to be opake, rough, and of a red mottled aspect.

We now come to the parts which were more immediately concerned in causing the death of the patient. McBride, as has been already seen, had been laboring for about three years under psoas abscess, with two fistulous apertures in the right lumbar region, and another in the left groin, immediately above Poupart's ligament. On tracing the latter of these, it was found to communicate with a large track, six inches long, by upwards of two in width, situated over the iliac and psoas muscles, and terminating above at the first lumbar vertebra. This passage was much narrower at the middle than at the extremities, and was lined throughout by a dense fibro-cartilaginous membrane, about the twelfth of an inch thick, and of a deep lilac hue, rough on the surface, and bathed with a thin, whitish, ropy pus. Superiorly, this passage communicated with several others, of which one inclined obliquely upwards and outwards in the direction of the inferior rib, where it ended in a sort of cul-de-sac; the rest terminated at the side of the lumbar vertebræ.

The other two apertures were situated very nearly on the same plane, about three inches from the spine. The upper, which was just beneath the last rib, and about one inch in diameter, after proceeding a short distance, terminated in a small, subcutaneous abscess, lined by a layer of lymph; the other, which was much smaller, and placed immediately above the iliac bone, communicated with a fistulous passage, eight inches in length, by two in width, which ran, like that on the opposite side, over the psoas and iliac muscles, commencing at Poupart's ligament, and stopping at the last dorsal vertebræ, gradually contracting in diameter. It was lined by a similar membrane, and bathed with a similar fluid.

The dorsal and lumbar vertebræ were nearly all carious in front, and at the sides bathed with pus, and most of the intervertebral cartilages and ligaments of the former destroyed. The periosteum was over a great extent of surface, and thickened with lymph. On the left side a pretty large abscess existed round the head of each rib, from the eighth to the twelfth, inclusive; they were filled with thick curdy matter, lined by an adventitious membrane, and most of them communicated with the corresponding intervertebral spaces.

This case beautifully illustrates the morbid anatomy of psoas abscess, and teaches how vain must generally be our best directed remedial efforts, when the disease depends upon caries of the spine.

### OPTHALMIC DEPARTMENT.

### Cataract.—Extraction.—Closed Pupil.

Case.—Mrs. E. C., of Indiana, admitted October 1st, 1836. Age 57. Constitution and health good. Cataract in both eyes, grey. Eyes not inflamed. The next day after administering a cathartic, Professor Drake proceeded, in presence of the pupils, to extract the lens from the right eye. The excision of the cornea was made without injury to any other part. At the proper time, and under a slight pressure of the eye ball, the lens did not advance, but sunk; and a portion of the vitreous humor escaped. The Professor was obliged to resort to the hook and culette, with which, at length, he succeeded in extracting it. On bringing it out, he discovered a partial adhesion of its capsule to the inner and inferior part of the iris. The capsule generally was decidedly vascular. The incision of the cornea healed up kindly; but a severe inflammation of the conjunctiva and iris supervened. It was met by abstinence, copious blood-letting, and a profuse salivation; but these measures were ineffective, and it was finally subdued by large doses of opium and tartar emetic. The cornea acquired a considerable degree of turbidness, on the disappearance of which, the pupil was found to be contracted, and closed with a plug of coagulable lymph. By mistake, the extract of belladonna had not been applied in due time. The eye gradually lost its morbid tenderness, its vascularity diminished, and on the 26th of November, the Professor undertook to make an artificial pupil. As there was already some opacity of the cornea from the first operation, Cheselden's knife was used, but in attempting to penetrate the iris from behind, it was found to be so tough, and thickened with layers of coagulable lymph, as to advance before the knife, till it came nearly in contact with the cornea. The incision which could be made was therefore partial, and no retraction of the iris took place. Perceiving that the operation would not be successful, the operator desired Professor Parker, (as the extraction of the lens had shown the cataract to be hard,) to couch the other eye. This he did immediately, but the lens arose so as to obstruct the lower part of the pupil.

A considerable degree of constitutional irritation and nausea, followed these operations, with pain in the eye balls and orbits. Dēnarcotised laudanum, and soda water, with the external application, by compresses, of a solution of belladona, afforded relief; but inflammation supervened and rendered bleeding, blistering, and the subsequent use of opium and tartar emetic necessary. By these means, she was able in three weeks to return home; having acquired a considerable degree of vision in the right eye, but without a pupil in the left. A late report of her case made at the Infirmary, represents the vision of that eye improving, although some part of the lens was still visible.

Clinical Observations.—At a subsequent time, Professor Drake made this case the subject of various comments.

He said, it might be thought that the severe inflammation which supervened, arose from the neglect of previous preparation of the patient's system; but such he did not believe was the fact. He has operated with and without such preparation, and seen no occasion to regard it as important. On the contrary, it is sometimes injurious, by rendering the patient's system irritable, and thus predisposing the eye to inflammation. The important point is, that the patient should be in health, and the eye free from inflammation; but when neither of these conditions existed, he would, after using due (though it might be unsuccessful) diligence, to restore sound general and local health, perform the operation. Wounds accidentally inflicted on those who are in bad health, often heal up kindly; and a chronic inflammation of the eye may be incurable. Should it be augmented by the operation, the patient cannot be said to be placed under circumstances of much greater difficulty; and may at length acquire a certain amount of vision, which will diminish his helplessness. As to the degree of inflammation, which may be expected after an operation for cataract, no means of judging *a priori*, exist. Now and then, it will be very small and very great, when the patient seems to have an equal chance of suffering or of immunity. It very often appears to depend on a state of the constitution, of which we cannot take cognizance. The professor has seen a very violent inflammation follow the introduction of the needle, both before and behind the iris, when that organ was not touched; and, at other times, has seen the extraction of the cataract followed by no more inflammatory action, than was necessary to adhesion. The operator, then, should never venture to predict a slight inflammation, and always be prepared for the worst.

On the remedies adapted to traumatic ophthalmia, the Professor remarked, that while venesection and local bleeding are generally indispensable, and in full and high toned systems, absolutely a *sine qua non* to success, they are far from being adequate to a cure, and are sometimes inadmissible, as a species of œdematous inflammation, without fever, occasionally supervenes. He is an advocate for calomel; but is assured by experience, that it will not always arrest the disease; and has, latterly, been led, in many cases, to the use of grain or even two grain doses of tartar emetic. But whether using the former or the latter, he thinks the union of opium, in large doses, greatly augments the efficacy of the prescription. In all severe cases of this kind, he would advise the following, every two or three hours, so as to keep the patient under the influence of the whole:

℞ *Opii. gr.* i. *Calomelan gr.* v. *Tart. Antimo gr.* i.—M. *fiat. pil* 1.

In some cases, the opium may be diminished to half a grain—in others the tartar may be augmented to two grains.

In referring to the difficulty of extracting the lens in this case, the Professor remarked, that there had evidently existed a chronic capsulitis, which had led to a partial posterior synechia. The vascular state of the capsule, taken in connexion with the adhesion, could leave no doubt on this point. Had depression been practised, the injury to the iris might have been less, but still, some degree of iritis would, in all probability, have followed the detachment of the lens from that membrane.

The pupil would not, probably, have closed, if the application of belladonna had not been inadvertently postponed to a late period. In speaking with regret on this point, the professor took occasion to say, that many cases of our endemic and infantile ophthalmia, are productive of closed pupil, from the omission of belladonna or some equivalent narcotic. The stramonium is in all respects equal to the belladonna for this purpose, and every country physician should be provided with it. A saturated tincture of the seeds will answer; or an extract may be formed from the leaves; or what is easier than either, and in some respects preferable, they may be stewed in fresh lard, which should be strained and decanted while hot. The extract of belladonna is often effete before it reaches the consumer, and always expensive compared with our native stramonium.

In the choice of the operation for forming an artificial pupil, the professor believed he had committed an error. The knife of Chesselden is, in fact, not adapted to cases in which the iris, covered with coagulable lymph, and rendered incapable of motion, cannot retract. It is difficult to cut it from behind, and the lips of the wound are apt to come together and unite. There is no certainty of success in such cases, but by removing a portion of the iris; to which end an incision through the cornea, as for the extraction of the lens, but of less extent, is required, when a portion of the iris may be drawn out with the hook, and cut off. Or the incision may be carried through the iris, as well as the cornea, when the scissors of Maunoir may be introduced, and by two cuts, a triangular portion of the iris removed, forming a pupil—much, it is true, to one side of the natural axis of vision, but still the best, perhaps, of which such a case permits. There are cases, the professor continued, to which this mode of operating is peculiarly adapted. They present the union of cataract and closed pupil, of which he gave the following example:

FORMATION OF ARTIFICIAL PUPIL AND EXTRACTION OF CATARACT.

CASE.—Several years since, a young man from Kentucky, applied to him with disorganization of the front of his right eye from inflammation, and contracted pupil and cataract of the left. The opake lens could be seen through the limited and undilatable pupil. The cornea and anterior chamber were well developed, and healthy. He had been blind for eleven years, and some distinguished surgeons had declined to operate upon him. Extraction of the lens and the excision of a part of the iris, at the same time, were determined on. The patient being placed as for the former operation, the knife was boldly plunged through both cornea and iris, and carried across the posterior disk of the latter, when it was brought out without touching the sclerotica, and the incision completed by the formation of two flaps. The aqueous humor of both chambers, of course, escaped simultaneously, and the solid opake lens followed almost immediately. There was, however, no loss of the vitreous humor, an accident which might be feared under such an excision. The cátaract being extracted, it only remained to secure an artificial pupil. To this end, the points of the scissors were carried across the flap, to the centre of the iris, and an incision made; it was to have been followed by another converging to the same centre, so as to take out a triangular piece, but retraction followed the first. No more inflammation supervened than was necessary to the adhesion of the edges of the cornea. No appreciable iritis occurred; the patient recovered rapidly; the aperture was large enough, and remained open. The professor at that time supposed the operation a new one, but has discovered, on more extensive reading, that it was not.

### Same operation unsuccessful.

Having been drawn thus far into the subject of artificial pupil, he would state another case, in which the result of the operation was very different.

Case.—Three or four years ago, a young man was brought to him from a workshop in Missouri, where by an explosion, the sight of both eyes had been destroyed. The cornea of the left eye had sloughed off; that of the right was partially opake and adherent near its margin (the upward especially) to the iris. The anterior chamber was, therefore, very small; the pupil was contracted and plugged up with a coagulum of lymph, or had the capsule of the lens adherent to it, which could not be determined. Presuming that factitious cataract existed, it was determined to operate as in the preceding case. The small extent of transparent cornea, rendered it necessary to cut through an opake part, and bring the knife out upwards. The incision being completed, through the iris as well as the cornea, a milky fluid (the lens) escaped. An attempt was then made to carry a blade of the scissors on either side of the iris; but an adhesion of that organ to the cornea (synechia anterior) had taken place, throughout the whole extent which had been traversed by the knife; and the outer blade of the scissors, could not be made to penetrate into the anterior chamber. The wound healed up kindly. Some time afterwards, the knife of Chesselden was pushed through the scelerotica, and with difficulty carried through the iris, which was pressed against the cornea, but no opening followed; and here again the pain and inflammation were very inconsiderable. Recourse was then had to Scarpa's curved cataract needle, which was carried across the narrow disk of the eye, and plunged through the iris, which could not, however, be detached from its ciliary ligament, because of the very limited space through which traction could be made. The formation of a pupil by coredialysis could not, therefore, be effected; but the iris was thick and inelastic from deposites of lymph, and the hole made in it remained open, forming a very small pupil. It could not be enlarged by the extract of belladonna, nor by the subsequent introduction of the needle. Now and then an object would happen to be placed before it, in such a position as to be seen, but for all practical purposes, the patient returned home as blind as he came.

After narrating this case, the professor called the attention of the class, to the little inflammation or constitutional irritation which followed these different operations; which he ascribed to the severe injury and inflammation following the accident that produced the blindness. The inflammation which then occurred, had transmuted the delicate modifications of the vital properties, which belong to the tissues of the eye, into an obtunded irritability and sensibility, and thus diminished the tendency to inflammation. The Professor has seen many examples of the same kind, as for instance, in repeated operations on the same eye, for the laceration of the cataract, he has uniformly observed every succeeding puncture to be attended with less constitutional irritation and inflammation than the preceding.

### Amaurotic Cat's Eye, mistaken for Cataract.

Case.—November 16, 1836, Miss H. W. was admitted into the Infirmary for blindness. Age about 22. Well grown, and general health tolerable, but suffers much pain in the forehead and orbits of the eyes. When about eight years of age, she had an attack of fever, in the course of which her right eye inflamed, and she found it painful to look in any other direction than downwards. The sight was lost; the eye retaining, as she stated, its natural appearance, except that the pupil was enlarged. About seven years afterwards, while she was running down a hill, a mist suddenly appeared before the left eye, and her vision became much impaired—and was gradually lost to such a degree, as that she had no distinct perception of objects. A more minute account of the progress of disease in this eye, could not be obtained. Being thus reduced to blindness, she applied to a physician, who decided on operating for cataract on the eye first affected. He introduced the needle behind the iris, but no improvement of sight ensued. The inflammation was severe, and was followed by closed pupil and some opacity of the cornea, which was the condition of her eye at the time of her admission into the Infirmary. Regarding this eye as lost, Professor Drake directed his attention to the other, with which the patient had a lively perception of the difference between day and night, and could even detect slight variations in the quantity of light, when the hand was passed between her eye and the window. The cornea was sound. The pupil regular in figure, but too large, and quite sluggish in its movements. Its color, under reflected light, inclined to slate. Such, as her friends stated, was precisely the appearance of the other eye previous to the operation. Professor D. directed the pupil to be enlarged to its utmost extent with belladonna, and then placed the patient in the sun. This direct light beautifully illumined the concave of the eye, which presented a pearly hue, with enlarged or varicose vessels, ramifying upon it. Under reflected light this appearance could not be seen.

The professor suggested, that a similar method might be employed in all cases, in which the diagnosis between cataract and amaurosis is difficult. He doubted the existence of black cataract; but, of course, it was possible. It could only be confounded with partial amaurosis. If the eye should be inspected by the direct rays of the sun, after the pupil was dilated, some change in the aspect of the choroid and retina would perhaps always appear, if the disease were amaurotic.

Jan. 12, 1837.—The patient returned to the Infirmary, anxious to be subjected to some remedial measures, especially electricity. Professor Parker, introduced a seton into her neck, and put her on the use of pills composed of equal parts of the blue mass, ipecac, and rhubarb, with the nitro-muriatic bath, to her hands and feet, and an occasional small bleeding. A blister was also applied to the left temple, and sprinkled every night with strichnine. Under this treatment, she thought her vision somewhat improved, and the pains about her forehead and temples were relieved. Electricity was then resorted to, and its use continued daily for several weeks. Slight shocks were passed through the orbit, and currents and sparks of electricity made to pass to and from the eye ball. No obvious benefit, however, came from this treatment, and she was discharged as incurable.

### Morbid Sensibility of the Eye.

Case.—On the 5th of May, 1837, J. E., aged 33, arrived at the Infirmary from a distant part of Indiana. He was brought in a wagon. His eyes were protected with deep green goggles. Before they were removed, he stated that within two years he had suffered several attacks of inflammation, the last of which, in the month of January, had been very severe, and left his eyes so "weak," that he could not bear the light, or keep them open, and that he feared he was going blind. "If Dr. Drake would not promise to cure him, he must go to Dr. Dudley, or to Philadelphia." Professor Drake advised him to proceed to Dr. Dudley; he asked, however, to have his eyes examined. On removing the goggles, no special morbid appearance was perceptible. The conjunctiva and iris appeared healthy; there was no puriform discharge, and the pupil had its natural play and dimensions. There was simply an inordinate sensibility to light. His general health appeared pretty good, except that he was costive. He had put on goggles during the attack of ophthalmia four months before. It was near sunset when he reached the Infirmary. Professor Drake ordered his goggles locked up, and directing a room for him, promised to decide on his case the next day, meanwhile administering a cathartic. In the morning, the light embarassed him, but at the prescribing hour, he was able to descend to the office, with no other shading than his hat. His eyes were reinspected, in presence of the pupils, and the professor gave the opinion, that it was only the morbid sensibility consequent upon the inflammation, *prolonged by the exclusion of light.* He was left to walk about the Infirmary, wearing his hat, and advised to expose his eyes to all the light he could bear without actual pain. On the second day, they were much "stronger." No local or general remedy was employed; but, much to his astonishment, he improved daily, and in a week started home quite restored.

Case.—In the month of June, a gentleman from Kentucky applied at the Infirmary for an alleged malady of the right eye. It was covered with a goggle lined and matted inside, so as entirely to exclude the light. The eye had been moderately inflamed, not long before. It now looked very well. Professor Drake laid the goggles by, and directed him to call daily. For a week he transacted business in the city with his eye exposed, and returned home apparently well.

Professor Drake observed on these cases, that he has repeatedly seen a morbid sensibility of one or both eyes, increased and prolonged, by the injudicious exclusion of light. He thinks the doctrine, that Mr. Stephenson, in his little work on Morbid Sensibility of the Eye, has labored to establish, is full of error. He admits, that there are cases of morbid sensibility dependent on inflammatory engorgement of the optic nerve and retina, but thinks they are few in number, compared with those which arise from the cause just mentioned. He presumes that had these patients been treated on Mr. Stephenson's plan of local depletion and other antiphlogistics, and light still excluded, they would have got worse instead of better. He has often seen that plan fail, and one precisely the opposite succeed; and referred briefly to the following

Case.—Several years ago, a woman, from Indiana, applied to him for "weak eyes." She wore deep green glasses, a shade, and, on going out, a bonnet and green veil. She had kept in a dark room for eighteen months. There was no visible inflammation. He subjected her to depletory treatment without effect; and then adopted the opposite, giving her the cinchona with chalybeates, and, gradually exposing her eyes to greater light. In a short time, she was able to walk half a mile to the steamboat, under a summer's sun, at noon, with nothing but a veil before her eyes, and returned home.

The Professor is of opinion that the curative influence of darkness in maladies of the eye, generally, has been over rated. He even thinks it has often done harm. The eye inevitably becomes irritable and unhealthy under such exclusion, which he compared to total abstinence from food, and every gastric excitant. He has seen the general health impaired by long continued confinement in a dark room; and the symptoms of ophthalmia thereby augmented, leading to increased energy in the use of antiphlogistics, when stimulants and tonics, with fresh air, and light were required. Whenever, in ophthalmia, stimulating collyria are proper, the eyes should be exposed to light. In concluding his clinical exhortations on this subject, the professor protested against the common custom among the people, of putting on green glasses, for every slight infirmity of the eye. Students, he remarked, often do themselves great injury by this practice. If the eye becomes disordered, let it be treated with energy, according to the nature and degree of the affection. This, whatever it may be, will seldom cease under the mere reduction of light; and as such reduction is often relied upon to effect a cure, it does harm by diverting the patient's attention from other and more efficent means.

